# Functional tumor-derived exosomes in NSCLC progression and clinical implications

**DOI:** 10.3389/fphar.2025.1485661

**Published:** 2025-03-19

**Authors:** Yuxin Gao, Jun Xie, Zhenya Yang, Mengxi Li, Hongfan Yuan, Rui Li

**Affiliations:** ^1^ Department of Abdominal Oncology, Sichuan Clinical Research Center for Cancer, Sichuan Cancer Hospital and Institute, Sichuan Cancer Center, University of Electronic Science and Technology of China, Chengdu, China; ^2^ Information Technology Center, West China Hospital of Sichuan University, Chengdu, China; ^3^ Information Technology Center, West China Sanya Hospital of Sichuan University, Sanya, China; ^4^ Xiangya School of Pharmaceutical Sciences, Central South University, Changsha, China; ^5^ College of pharmacy, Chengdu Medical College, Chengdu, China; ^6^ Department of Oncology, Sichuan Clinical Research Center for Cancer, Sichuan Cancer Hospital and Institute, Sichuan Cancer Center, University of Electronic Science and Technology of China, Chengdu, China; ^7^ Department of Radiation Oncology, Radiation Oncology Key Laboratory of Sichuan Province, Sichuan Clinical Research Center for Cancer, Sichuan Cancer Hospital and Institute, Sichuan Cancer Center, University of Electronic Science and Technology of China, Chengdu, China

**Keywords:** exosomes, NSCLC, tumor progression and metastasis, tumor drug resistance, immune regulation

## Abstract

Non-small cell lung cancer (NSCLC) accounts for approximately 85% of all lung cancer cases and remains one of the leading causes of cancer-related mortality worldwide. The high mortality rate is primarily driven by delayed diagnosis, rapid metastasis, and frequent recurrence. Tumor-derived exosomes (TEXs) have emerged as critical mediators in NSCLC progression, offering valuable insights into the tumor microenvironment. Exosomes are small membrane vesicles that facilitate intercellular communication and transport bioactive molecules, including proteins, RNAs, and DNAs, thereby reflecting the genetic complexity of tumors. These exosomes play a key role in promoting tumor metastasis, epithelial-mesenchymal transition (EMT), neovascularization, drug resistance, and immune evasion, all of which are pivotal in the development of NSCLC. This review explores the diverse roles of TEXs in NSCLC progression, focusing on their involvement in pre-metastatic niche formation, tissue metastasis, and immune modulation. Specifically, we discuss the roles of exosome-associated RNAs and proteins in NSCLC, and their contribute to tumor growth and metastasis. Furthermore, we explore the potential of TEXs as biomarkers for NSCLC, emphasizing their application in diagnosis, prognosis, and prediction of resistance to targeted therapies and immunotherapies.

## 1 Introduction

NSCLC is the most common type of lung malignancy, accounting for approximately 80%–85% of all diagnosed lung cancer cases (Reck et al., 2022). Over the past decade, the incidence of NSCLC has declined from 46.4 to 40.9 per 100,000 cases, while the incidence in patients under 65 years old has shown an upward trend. Moreover, over 60% of patients are diagnosed at an advanced stage, with a 5-year survival rate falling below 15% (Siegel et al., 2022). Epidemiological studies have shown that approximately 40% of NSCLC patients experience recurrence after surgery (Han B. et al., 2022), and the 5-year survival rate for metastatic NSCLC patients is 7% (Rizwan et al., 2022). These statistics emphasize the urgent need for improved early diagnostic accuracy and enhanced treatment monitoring to improve prognosis and survival outcomes. Hence, identifying critical molecular players in NSCLC progression and metastasis, as well as discovering highly sensitive and specific biomarkers, is crucial for early diagnosis, prognostic assessment, and predicting treatment responses.

Approximately 20% of cancer patients are unable to undergo tissue biopsy due to patient conditions, technical limitations, or the inherent heterogeneity of tumor tissues, which hampers diagnostic accuracy (Rolfo et al., 2021; Shields et al., 2022). Non-invasive diagnostic methods, especially liquid biopsy, may become powerful tools for tumor diagnosis and identifying tumor biomarkers (Bertoli et al., 2023; Ma et al., 2024). Liquid biopsy enables the isolation and analysis of circulating cell-free DNA, RNA, and proteins from blood or other body fluids in cancer patients, offering a simplified, more convenient, and better-tolerated diagnostic method (Levy et al., 2016; Molina-Vila et al., 2016).

Exosomes, classified as members of the extracellular vesicle family, were firstly recognized in the early 1980s as vesicles (Nazarenko, 2020; Zhang Y. et al., 2019). These vesicles are released by a wide variety of normal and malignant cells (Whiteside TheresaL., 2016; Zhang et al., 2015). Exosomes have been successfully isolated and purified from various bodily fluids, including blood, encompassing urine, saliva, pleural effusion, ascites, breast milk, and bronchoalveolar lavage fluid, which have potential application prospects in liquid biopsy (Conde-Vancells et al., 2008). Recent research has primarily focused on the roles of exosomes in tumor diagnosis, disease monitoring, and evaluating treatment efficacy (Padinharayil and George, 2024; Ren et al., 2024). Exosomes also play pivotal roles in carcinogenesis and tumor progression, including intercellular signaling, metastasis, drug resistance, and immune suppression (Ren et al., 2024; Hamid et al., 2025; Li Dongqi et al., 2024). Our review highlights the critical role of exosomes in NSCLC progression and metastasis, while exploring their potential as non-invasive biomarkers for early detection and disease monitoring.

## 2 The functions of TEXs

Exosomes are active nanovesicles, composed of lipid bilayers, with diameters ranging from 40 to 150 nm. They originate from multivesicular bodies and are released into the extracellular space through fusion with the plasma membrane of various normal and tumor cells (Gurung et al., 2021; Liu et al., 2020; Daßler-Plenker et al., 2020). Initially, exosomes were believed to function solely as cellular waste disposal units, responsible for eliminating unwanted molecules within cells (Pan et al., 1985). However, recent studies have demonstrated that these vesicles play more complex roles, including transmitting biological information to neighboring cells and significantly contributing to carcinogenesis and tumor progression (Li Junshu et al., 2024; Agrawal et al., 2024; Oh et al., 2024). Exosomes facilitate the exchange of genetic material via autocrine, paracrine, and endocrine pathways within the cellular environment (Krylova and Feng, 2023; Arya et al., 2024). They deliver their contents through three primary mechanisms: fusion with the plasma membrane, resulting in the release of their internal contents; endocytosis; and interaction with cell surface receptors (Krylova and Feng, 2023). Thus, exosomes are considered a novel mode of cellular signaling. Exosomes can be identified using techniques such as nanoparticle tracking analysis (NTA), resistance pulse sensing (RPS), transmission electron microscopy (TEM), and flow cytometry (FCM) (Yuana et al., 2013; Xie et al., 2019). Methods for isolating and purifying exosomes include ultrafiltration, ultracentrifugation, immunoprecipitation, precipitation, and density gradient centrifugation (Xie et al., 2019; Mortezaee et al., 2022). The contents of exosomes vary depending on the type of secreting cell and include DNA, RNA, proteins, metabolic products, lipids, and cell membrane proteins (Kalluri and LeBleu, 2020; van der Pol et al., 2012; Najafi, 2022).

In normal human blood, approximately 200 trillion exosomes can be identified, whereas the blood of cancer patients contains approximately 400 trillion exosomes (Kalluri, 2016). TEXs have been successfully isolated from various bodily fluids, including urine, saliva, pleural effusion, ascites, breast milk, and bronchoalveolar lavage fluid (Conde-Vancells et al., 2008), highlighting the propensity of cancer cells to produce exosomes in higher concentrations. This suggests their potential as innovative tumor biomarkers. Furthermore, TEXs play a crucial role in the progression of malignant tumors and the development of distant metastases. TEXs regulate the tumor microenvironment, promote angiogenesis and EMT, enhance intercellular signaling, increase tumor cell invasiveness, and foster the establishment of a pre-metastatic niche that promotes distant metastasis (Ren et al., 2024; Hamid et al., 2025). Notably, TEXs influence immune regulation by affecting intercellular communication, immune activation, immune surveillance, antigen expression, and immune suppression (Greening et al., 2015; Dong et al., 2025). TEXs can also carry tumor-associated antigens, potentially impairing the efficacy of immunotherapy (Whiteside TheresaL., 2016). In addition, exosomes serve as key mediators in the resistance signaling pathways of malignant tumor cells, facilitating the transmission of resistance signals in response to targeted therapies (Zhang et al., 2015). Thus, TEXs may hold significant clinical potential for guiding diagnosis, predicting metastasis, evaluating treatment response, and understanding resistance mechanisms in malignant tumors.

## 3 Related exosome factors involved in NSCLC progression and metastasis

In malignant tumors, exosomes play a pivotal role in tumor progression and metastasis by modulating immune responses, promoting angiogenesis and influencing EMT (Ridder et al., 2015; Can et al., 2025). TEXs facilitate the evasion of immune surveillance by transferring specific proteins to recipient cells, thereby altering the functional behavior of immune cells and promoting tumor progression (Whiteside TheresaL., 2016). For instance, heat shock protein 72 (HSP72) carried by TEXs enhances the immunosuppressive capability of myeloid-derived suppressor cells (MDSCs) via a STAT3-dependent pathway, contributing to immune tolerance of tumor cells (Chalmin et al., 2010). TEXs also inhibit T cell proliferation and induce apoptosis by activating the FAS/FASL signaling pathway, exerting an immunosuppressive effect (Kim et al., 2005). Additionally, TEXs suppress natural killer (NK) cell activity and interfere with monocyte differentiation (Whiteside T. L., 2016). Tumor exosomes can also deliver epidermal growth factor receptor (EGFR) to host macrophages, inhibiting the production of type I interferons and thereby reducing the overall immune response in cancer patients (Gao et al., 2018).

Angiogenesis is critical for providing the blood supply necessary for tumor growth and metastasis (Goudar and Vlahovic, 2008). In the peripheral tissues surrounding malignant tumors, an equilibrium exists between pro-angiogenic and anti-angiogenic factors, which regulate the angiogenesis process. However, malignant tumors are characterized by a predominance of pro-angiogenic proteins, leading to the promotion of neovascularization (Olejarz et al., 2020; Liu et al., 2023). Tumor-derived angiogenic factors, along with other components in the tumor microenvironment, stimulate the proliferation and migration of endothelial cells, thereby facilitating new blood vessel formation to meet the tumor’s nutritional demands (Gasparics and Sebe, 2022).

EMT involves biochemical changes in epithelial cells, leading to the acquisition of mesenchymal phenotypes characterized by increased migratory and invasive capacities, as well as elevated production of extracellular matrix (ECM) components (Gasparics and Sebe, 2022; Greco et al., 2022; Zhang et al., 2022). EMT facilitates tumor metastasis by reducing intercellular adhesion among differentiated epithelial cells, allowing tumor cells to move either individually or collectively (Zhang et al., 2022; Han J. et al., 2022; Kim, 2022). Exosomes have been shown to contribute to both neovascularization and EMT in tumor cells (Lin et al., 2022; Song et al., 2022; Amicone et al., 2022). Here, we summarize the exosomal signaling factors involved in regulating the progression and metastasis of NSCLC.

### 3.1 Role of exosomal RNA in promoting cancer progression in NSCLC

Exosomal RNA is produced via the endocytosis process within the cell and primarily consists of three distinct classes of non-coding RNA: microRNAs (miRNAs) (Kogure et al., 2011), long non-coding RNAs (lncRNAs) (Lee et al., 2017; Min et al., 2018), and circular RNAs (circRNAs) (Andrey et al., 2011). Notably, studies have shown significantly higher expression levels of exosomal RNA in cancer patients compared to healthy individuals (Tang et al., 2024; Yi et al., 2024; Yue et al., 2024). These exosomal RNAs play pivotal roles in regulating key processes involved in tumor progression, including immune modulation, angiogenesis, metastasis, and drug resistance, contributing to the overall dynamics of the tumor microenvironment.

MiRNAs are a class of non-coding RNA (ncRNA) molecules, approximately 22 nucleotides in length, that regulate gene expression by binding to the 3′untranslated region or open reading frame of target messenger RNA (mRNA) (Saliminejad et al., 2019). In NSCLC, specific miRNA profiles are closely associated with tumor behavior and treatment response, and these miRNAs can be clinically detected by extracting exosomes from body fluids (Shanehbandi et al., 2023; Janpipatkul et al., 2021). Tumor cells release distinct miRNAs into the extracellular space, which are transported via exosomes circulating in the bloodstream (Du et al., 2018). Moreover, tumor cells produce exosomes in particularly high quantities under hypoxic conditions, where they play a crucial role in angiogenesis (Sandúa et al., 2021; Meng et al., 2019). Under hypoxic conditions, exosomes released by lung cancer cells show a significant upregulation of *miR-23a*, leading to the accumulation of hypoxia-inducible factor-1α (HIF-1α) in endothelial cells. This, in turn, increases tumor angiogenesis and vascular permeability, thereby promoting metastasis (Hsu et al., 2017). Another study also found that *miR-619-5p* is transferred to exosomes from NSCLC cells under hypoxic conditions, promoting tumor angiogenesis by inhibiting *RCAN1.4* (Kim et al., 2020). Additionally, *miR-3157-3p* is transported from NSCLC cells to vascular endothelial cells through exosomes, targeting the vascular endothelial growth factor (VEGF)/matrix metalloproteinase 2 (MMP2)/MMP9 pathway to enhance the formation of new blood vessels (Ma et al., 2021). Additionally, *miR-3157-3p* is transported from NSCLC cells to vascular endothelial cells through exosomes, targeting the VEGF/MMP2/MMP9 pathway to enhance the formation of new blood vessels (Gu et al., 2021). Exosomal *miR-34c-3p* upregulates integrin α2β1, enhancing the invasive and migratory capacities of NSCLC cells (Huang et al., 2020).

LncRNAs are a class of RNA molecules that exceed 200 nucleotides in length (Bridges et al., 2021). Exosome-associated lncRNAs play key roles in tumor progression by regulating metastasis, stem cell maintenance, drug resistance, and the tumor microenvironment (Lin et al., 2023). One of the most studied lncRNAs in NSCLC is the metastasis-associated lung adenocarcinoma transcript 1 (*MALAT1*), which is highly expressed in the serum of NSCLC patients and promotes tumor migration by inhibiting apoptosis and shortening the cell cycle (Zhang et al., 2017). Elevated levels of *lnc-UFC1* have been detected in the exosomes of NSCLC patient serum, and the increase in *UFC1* levels is associated with NSCLC invasion (Zang et al., 2020). Another study found that exosomes in the plasma of metastatic NSCLC patients show elevated levels of the lncRNAstem cell inhibitory RNA transcript (*SCIRT*), which is linked to survival in metastatic NSCLC (Wang et al., 2021). Interestingly, lncRNA *SCIRT* does not directly promote cancer progression but selectively sorts *miR-665* into TEXs in a *hnRNAPA1*-dependent manner. Subsequently, exosomes enriched with *miR-665* directly impact the enhancement of NSCLC invasion and migration capabilities by targeting the Notch downstream transcription factor *HEYL*. Furthermore, exosomal lncRNAs such as *HOTAIR* (Chen L. et al., 2021) and *NEAT1* (Hussain et al., 2024), frequently upregulated in NSCLC, regulate cellular responses to external stimuli like hypoxia and oxidative stress, common in the tumor microenvironment. By acting as scaffolds for chromatin-modifying complexes, these lncRNAs promote tumor progression and chemoresistance. Targeting these lncRNAs in exosomes could provide a novel strategy to prevent metastasis and improve treatment outcomes in NSCLC.

CircRNAs are formed by back-splicing and have a unique covalent closed-loop structure, providing stability within cells and enabling them to regulate gene expression and affect biological functions (Zhou et al., 2020). *circSATB2* promotes the progression of NSCLC and is upregulated in serum exosomes derived from cancer patients. Serum exosomal *circSATB2* in patients with metastatic cancer suggests its potential role as a tumor biomarker for NSCLC (Zhang N. et al., 2020). Exosomes secreted by NSCLC repress the function of CD8^+^ T cells and contribute to resistance to anti-programmed cell death protein-1 (anti-PD1)immunotherapy (Chen et al., 2021b). In addition to *circSATB2*, other circRNAs, such as *circ_0001946* (Zhang et al., 2021), *circPVT1* (Huang et al., 2021), and *circHIPK3* (Siedlecki et al., 2024), have also been associated with NSCLC. These circRNAs contribute to the initiation and progression of lung cancer by modulating various signaling pathways and gene expressions. They show promise as potential biomarkers for early diagnosis, prognosis assessment, and targeted therapies.

### 3.2 The functional proteins exosomes in NSCLC

In addition to ncRNAs, exosome proteins have also been considered as key molecules mediating the metastatic phenotype of NSCLC cells. Exosome proteins are mainly membrane transport and fusion proteins, such as annexins, RAB5/RAB7, and TSG101 (Théry et al., 2002). Among the most widely recognized exosome membrane proteins are the tetraspanins, including CD9, CD63, and CD81 (Rana and Zöller, 2011), which are overexpressed on the surface of exosomes and regulates cell-cell interactions, thereby influencing tumor behavior and progression (Zhang W. et al., 2019). These proteins facilitate the exchange of cellular information between the tumor and the surrounding microenvironment, aiding in metastasis and immune evasion.

A key protein involved in NSCLC metastasis is hepatocyte growth factor (HGF), which is enriched in exosomes derived from highly metastatic tumor cells. Exosomal HGF plays a pivotal role in promoting EMT and facilitating cancer cell migration and invasion. It achieves this by activating the c-Met receptor on non-metastatic cells, thereby triggering downstream signaling pathways that drive metastasis (Qiao et al., 2019). Similarly, exosomal proteins derived from the serum of patients with advanced malignancies have been shown to increase the expression of vimentin (VIM) and enhance the metastatic phenotype in recipient cells. This suggests that exosome-mediated protein transfer plays a significant role in promoting the EMT process, which is essential for metastasis (Rahman et al., 2016).

Exosomal proteins also contribute to tumor progression by modulating the immune response. For instance, exosomes secreted by NSCLC cells can interfere with CD8^+^ T cell function, promoting immune evasion (Chen et al., 2021c). These exosomes carry proteins that inhibit T cell activation and cytotoxicity, allowing the tumor to escape immune surveillance. This mechanism contributes to the resistance of NSCLC to immunotherapy, such as anti-PD1 treatments, by dampening the immune response against tumor cells (Rahman et al., 2016). In addition, exosomes facilitate tumor angiogenesis by delivering pro-angiogenic factors such as VEGF and MMPs to endothelial cells. The transfer of EGFR via exosomes to endothelial cells activates the mitogen-activated protein kinases (MAPK) and protein kinase B (AKT) signaling pathways, which, in turn, upregulate VEGF expression and enhance blood vessel formation (Al-Nedawi et al., 2009).

In conclusion, exosomal proteins in NSCLC play a central role in facilitating tumor progression, metastasis, and immune evasion. By influencing various signaling pathways, these proteins promote the transition from localized tumor growth to widespread metastatic disease. As such, exosomal proteins have significant potential as biomarkers for NSCLC diagnosis and prognosis, as well as therapeutic targets for inhibiting metastasis and enhancing the effectiveness of immunotherapies.

## 4 Exosome-mediated pre-metastatic niche formation and tissue metastasis

The target organs for malignant tumor metastasis are not selected randomly. In 1889, the “seed and soil” hypothesis was proposed, suggesting that certain tumor cells, referred to as “seeds,” have a propensity to metastasize to specific organs, termed as “soil.” Tumor cells can only successfully metastasize when the environment, or “soil,” is conducive to their growth (Paget, 1889). Despite extensive research, the specificity of organ targeting in tumor metastasis remains one of the most profound mysteries in the field. Recent studies have shown that exosomes play a crucial role in this process, facilitating the establishment of pre-metastatic niches before direct contact between the primary tumor and the distant organ (Milane et al., 2015; Su et al., 2021).

**FIGURE 1:**
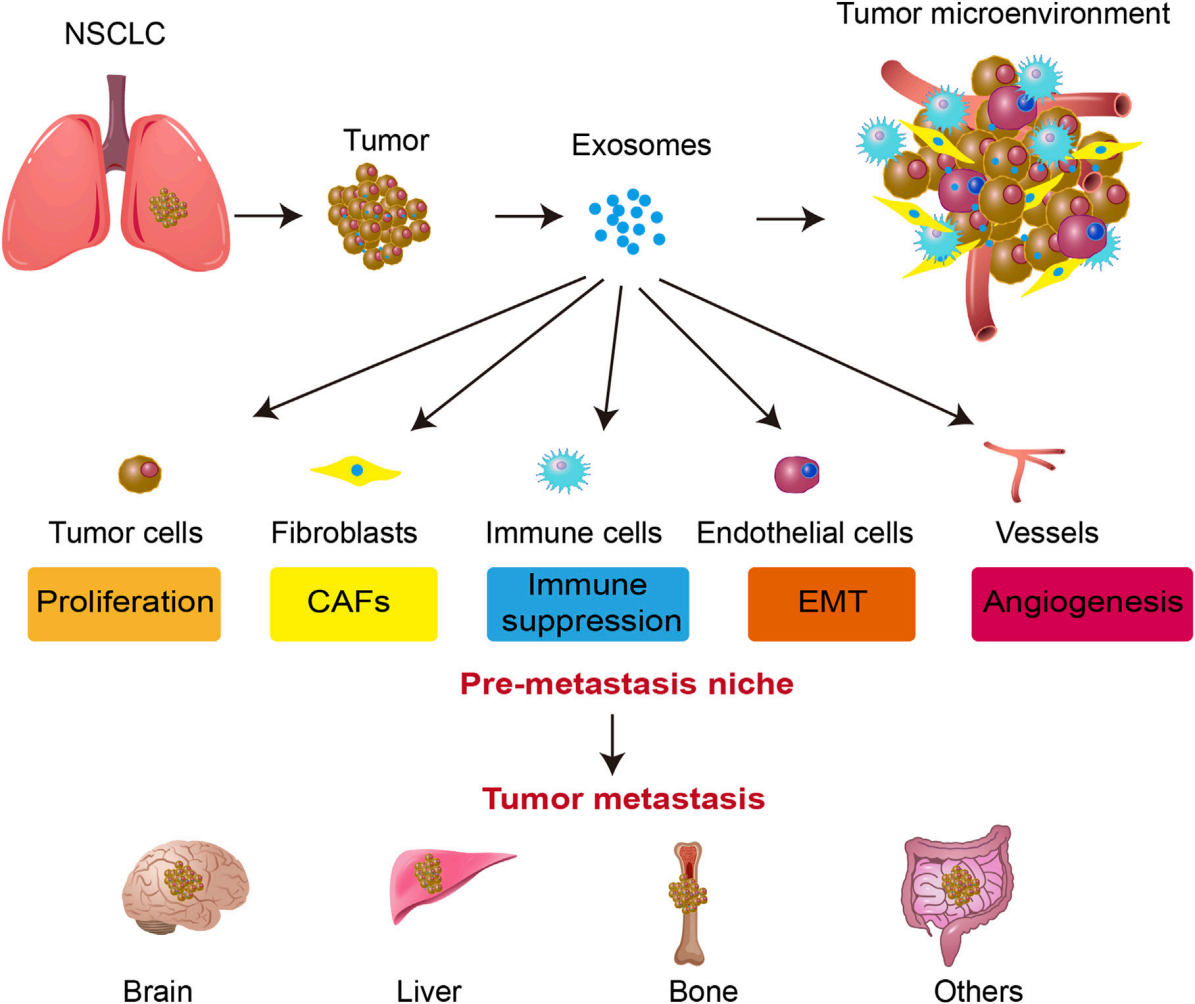
Exosome-mediated tumor progression and metastasis in NSCLC. Exosomes secreted by tumor cells influence various components of the tumor microenvironment, including fibroblasts (CAFs), immune cells, endothelial cells, and blood vessels. Exosomes facilitate tumor cell proliferation, immune suppression, EMT, and angiogenesis. These processes contribute to the formation of a pre-metastatic niche, ultimately leading to tumor metastasis to distant organs such as the brain, liver, and bone. The figure highlights the critical role of exosomes in promoting NSCLC progression and metastatic spread.

Exosomes facilitate tumor metastasis through various mechanisms. They contribute to the establishment of pre-metastatic niches by transferring molecular signals that prime distant organs for tumor cell colonization. Additionally, exosomes promote EMT, enhancing tumor cell motility and invasiveness. They also play a crucial role in angiogenesis and increase vascular permeability, facilitating tumor cell dissemination via the bloodstream. Moreover, exosomes contribute to immune modulation by suppressing antitumor immune responses, thus enabling immune evasion (Luo et al., 2023; Zhao et al., 2021). These coordinated actions enhance the metastatic potential of tumor cells, supporting their colonization in secondary sites (Figure 1).

The organotropism of different types of metastatic tumor cells showed significant differences (Obenauf and Massagué, 2015), which are related to the integration of TEXs (Hoshino et al., 2015a). Proteomic analysis reveals that exosomes isolated from tumor cells originating from distinct organs exhibit distinct integrin expression patterns. Specifically, integrin *α6β4* and *α6β1* are associated with lung metastasis, whereas integrin *αvβ5* is correlated with liver metastasis. The disruption of integrin *α6β4* and *αvβ5* expression has been shown to diminish the uptake of exosomes by recipient organ cells, thereby reducing lung and liver metastasis, respectively (Hoshino et al., 2015b).

The main sites of NSCLC metastasis are the bone, brain, and liver (Wood et al., 2014). Recent research has conclusively demonstrated the pivotal role that exosomes play in establishing a pre-metastatic immune microenvironment conducive to brain metastasis. The biological distribution of exosomes secreted by tumors was analyzed, found a tissue-specific fusion of integrins with T cells, which in turn facilitates organ-specific colonization and the formulation of a pre-metastatic niche tailored specifically for brain metastasis (Xu et al., 2020). In addition, *CEMIP +* exosomes secreted by tumors are absorbed by brain cells and microglia, leading to the enhanced expression of pro-inflammatory cytokines, which are encoded by genes such as *PTGS2*, *TNF*, and *CCL/CXCL*, thereby promoting cerebrovascular remodeling and metastasis (Rodrigues et al., 2019).

Additionally, exosomes derived from NSCLC cells treated with transforming growth factor (TGF-β) contain high levels of *lnc-MMP2-2.* This lncRNA stimulates MMP2 expression, positively correlating with tumor cell invasiveness and vascular permeability, further promoting metastasis (Wu et al., 2018; Valadi et al., 2007; Liao et al., 2015; Chen et al., 2013; Tang et al., 2016). A recent investigation has revealed that *lnc-MMP1-2* disrupts the tight junctions present between human brain microvascular endothelial cells. Additionally, it has been observed to induce EMT and enhance the permeability of the blood-brain barrier, allowing tumor cells to penetrate the brain in the circulatory system (Wu et al., 2021).

Except for brain metastasis, bone metastasis is also a prevalent form of distant metastasis observed in NSCLC. Exosomes extracted from peripheral blood of NSCLC patients with bone metastasis exhibit a significant upregulation of SOX2 overlapping transcript (*SOX2-OT*), which is closely associated with lower overall survival rates. *SOX2-OT* increases *RAC1* expression by targeting *miR-194-5p* to promote bone metastasis of NSCLC (Ni et al., 2021).

## 5 Exosomes as biomarkers of NSCLC

Conventional biomarkers, such as carcinoembryonic antigen (CEA), epithelial cell adhesion molecules (EPCAM), and EGFR, can be found in lung tissue, tumor-draining pulmonary blood, and bone marrow samples used for diagnosing NSCLC. However, these techniques are challenging to samples and cause much discomfort to patients (Rehulkova et al., 2023; Mederer et al., 2022). “Liquid biopsy” represents a non-invasive or minimally invasive approach to disease detection, utilizing molecular diagnostic techniques as its foundation (Ma et al., 2024). Recently this technology, which has become a research hotspot, utilizes bodily fluids such as blood, bronchial alveolar fluid, urine, pleural effusion, ascites, breast milk, and saliva from cancer patients to detect circulating biomarkers of tumors and obtain relevant genetic information about the disease (Buszka et al., 2022). It possesses the capability to identify tumors at an earlier stage than imaging techniques, rendering it a suitable tool for the early diagnosis of tumors.

**FIGURE 2:**
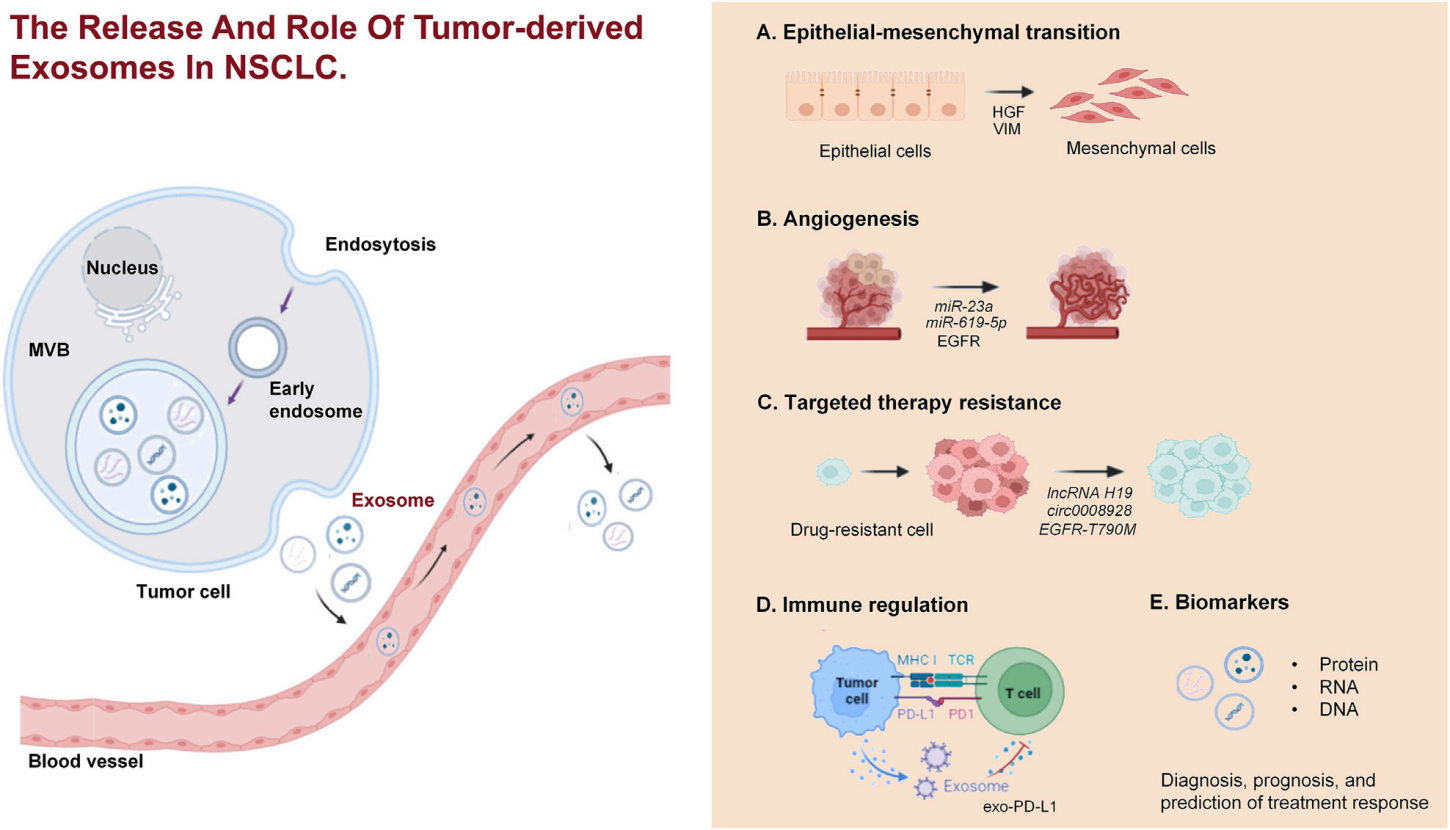
The release and role of TEXs in NSCLC. TEXs in NSCLC are released from tumor cells via the endocytic pathway, involving the formation of early endosomes, which mature into multivesicular bodies. These MVBs then fuse with the plasma membrane, releasing exosomes into the extracellular environment. The exosomes contribute to various processes in NSCLC, including EMT, angiogenesis, drug resistance, immune regulation, and they serve as biomarkers for diagnosis, prognosis, and treatment response prediction.

In the era of liquid biopsy, using exosomes as biomarkers for NSCLC is a promising approach. Exosomes, which are directly secreted into bodily fluids by tumor cells, encompass components such as ncRNAs and protein alterations (including EGFR mutations), thereby rendering highly distinctive and representative information (Casagrande et al., 2023; Vasu et al., 2025). Exosomes exhibit a ubiquitous distribution, possess remarkable permeability, are easily accessible, and are encapsulated by different lipid bilayers and are not easily degraded (Cheng et al., 2022; Boukouris and Mathivanan, 2015). The identification of exosomes with differential expression patterns in liquid biopsy exhibits promising applications in various medical domains, including the diagnosis of cancer, prognostic evaluation, monitoring of disease progression, and assessment of chemotherapy resistance (Figure 2) (Rezaie et al., 2022). We have conducted a comprehensive summary of extracellular vesicle RNA and protein as potential biomarkers for the diagnosis, prognosis, and prediction of treatment response in NSCLC, as presented in Tables 1–4.

**TABLE 1:** Exosomal miRNAs as biomarkers for NSCLC.

Exosomal miRNAs	Source	Expression	Clinical value	AUC	References
miR-3182	serum	upregulation	differentiating early-stage NSCLC patients from those with benign pulmonary diseases	0.785	Visan et al. (2022)
miR-1290	plasma	upregulation	early lung cancer diagnosis, distinguishing between NSCLC and SCLC	0.947	Zhang et al. (2023)
miR-29c-3p		downregulation		0.895	
miR-1169	serum	upregulation	differentiating NSCLC carrying wild-type EGFR	1.000	Han et al. (2020)
miR-260			differentiating NSCLC patients carrying mutant EGFR	0.997	
miR-let-7f-5p	plasma	downregulation	diagnosing NSCLC with a combination of CEA and Cyfra 21-1	0.981	Wang et al. (2020)
miR-128-3p	serum	downregulation	NSCLC diagnosis combined with miR-128-3p	0.855	Li et al. (2023)
miR-1260b	plasma	upregulation	associated with poorer survival		Kim et al. (2021)
miR-320	plasma	upregulation	predicting disease progression with PD-1/PD-L1 inhibitor treatment		Peng et al. (2020b)
miR-125b-5p					
miR-125a-3p	serum	upregulation	predicting disease progression with PD-1/PD-L1 inhibitor treatment		Hisakane et al. (2023)
miR-433	plasma	downregulation	low expression in the plasma of resistant NSCLC patients, negatively correlated with tumor size, distant metastasis, advanced TNM staging, and poor prognosis		Liu et al. (2021)
miR-4497	serum	downregulation	identifying tumor size, TNM staging, and distant metastasis	0.895	Zheng et al. (2023)
miR-1258-3p miR-17-5p miR-184 miR-3913-5p miR-323-3p miR-1468-3p miR-5189-5p miR-6513-5p miR-494-3p	plasma	upregulation	discerning osimertinib-resistant from osimertinib-sensitive NSCLC patients		Janpipatkul et al. (2021), Han et al. (2023), Kaźmierczak et al. (2022)

**TABLE 2:** Exosomal lncRNAs as biomarkers for NSCLC.

Exosomal lncRNAs	Source	Expression	Clinical value	AUC	References
linc01125	serum	upregulation	diagnosing early-stage lung cancer	0.662	Xian et al. (2021)
RP5-977B1	serum	upregulation	diagnosis and prognostic assessment of early-stage NSCLC	0.889	Min et al. (2022b)
LINC00917	serum	upregulation	diagnosing patients with stage III/IV NSCLC	0.907	Xiong et al. (2021)
lncCRLA	plasma	upregulation	diagnosing early-stage lung adenocarcinoma		Lin et al. (2024b)
SNHG15	serum	upregulation	distinguishing NSCLC patients from healthy individuals	0.856	Han et al. (2021)
			diagnosing stage I/II NSCLC patients	0.838	
			diagnosing stage III/IV NSCLC patients	0.870	
			NSCLC diagnosis combined with CEA	0.915	
PGM5-AS1, SFTA1P, CTA-384D8.35D8.35	plasma	upregulation	NSCLC diagnostic model	0.97	Wang et al. (2023)
lncRNA-GHSROS, lncRNA-HNF1A-AS1, lncRNA-HOTAIR	serum	upregulation	diagnosis of NSCLC	0.947	Talebi et al. (2022)
lncRNA-P21, lncRNA-HMlincRNA717		downregulation			
SOX2OT	plasma	upregulation	enriched in peripheral blood exosomes of NSCLC patients with bone metastasis, associated with shorter overall survival		Ni et al. (2021)
HOTAIR	serum	upregulation	significantly correlated with lymph node metastasis and TNM staging	0.821	Chen et al. (2021a)
lnc-SNAPC5-3:4	plasma	downregulation	monitoring resistance to anlotinib treatment		Liu et al. (2022)

**TABLE 3:** Exosomal circRNAs as biomarkers for NSCLC.

Exosomal circRNAs	Source	Expression	Clinical value	AUC	References
circ_0069313	serum	upregulation	differentiating benign pulmonary tumors from NSCLC, associated with stage III-IV NSCLC, lymph node metastasis, and distant metastasis	0.749	Chen et al. (2022b)
circ_ERBB2IP	serum	upregulation	related to TNM staging, lymph node metastasis, and tumor size in NSCLC patients	0.917	Peng et al. (2023)
circ_102481	serum	upregulation	significantly upregulated in NSCLC with resistance to EGFR-TKIs, associated with TNM staging, tumor differentiation status, brain metastasis, and survival		Yang et al. (2021)
circ_0008928	serum	upregulation	upregulation in the serum exosomes of cisplatin-resistant NSCLC patients		Shi et al. (2023)
circ_VMP1	serum	upregulation	upregulation in the serum exosomes of cisplatin-resistant NSCLC patients		Xie et al. (2022)
circ_KIF20B	serum	downregulation	low expression in NSCLC patients resistant to Gefitinib, negatively correlated with tumor size and staging		Wei et al. (2023)

**TABLE 4:** Exosomal proteins as biomarkers for NSCLC.

Exosomal proteins	Source	Clinical value	AUC	References
lipopolysaccharide-binding protein	serum	differentiating metastatic from non-metastatic NSCLC patients	0.803	Wang et al. (2018)
multifunctional glycoproteins	plasma	predicting the genesis of NSCLC, diagnosing NSCLC	0.732	Chang et al. (2023)
		utilizing a mix of plasma and plasma exosomes’ multifunctional glycoproteins to diagnose NSCLC	0.804	
IGHV4-4, IGLV1-40	plasma	diagnosis of NSCLC	0.93	Yang et al. (2023)
		differentiating between metastatic and non-metastatic NSCLC	0.88	
AHSG, ECM1	serum	combining with CEA for the diagnosis of early-stage NSCLC	0.938	Niu et al. (2019)
PD-L1	serum	associated with a poorer prognosis		Shimada et al. (2021)
DOK3	plasma	associated with favorable prognosis in Gefitinib treatment		Ochiai et al. (2022)
MFGE8	plasma	differentiating between lung squamous cell carcinoma and lung adenocarcinoma		Bao et al. (2022)

### 5.1 TEXs as biomarkers for NSCLC diagnosis

Owing to the enhanced production of exosomes by tumor cells, exosomes present a promising role as novel diagnostic biomarkers. The diagnostic potential of exosomes in plasma or serum of NSCLC patients can be determined by analyzing the area under the gene expression curve (AUC). Exosomal *miR-3182* (Visan et al., 2022), *miR-1290*, and *miR-29c-3p* (Zhang et al., 2023) have been shown to be useful in the early detection of lung cancer. In comparison to conventional tumor biomarkers, exosomal *miR-1290* and *miR-29c-3p* exhibit superior diagnostic efficacy in discerning benign lung diseases from lung cancer, achieving AUC values of 0.934 and 0.868 respectively. These miRNAs demonstrate higher diagnostic accuracy for early-stage lung cancer, with AUC values of 0.947 and 0.895, compared to traditional markers (Zhang et al., 2023). Circulating exosomal *miR-342-5p* and *miR-574-5p* were significantly elevated in early-stage lung adenocarcinoma (LA) patients compared to healthy controls and decreased after tumor resection. The combination of these miRNAs achieved an AUC of 0.813, with 80% sensitivity and 73.2% specificity, underscoring their potential as novel biomarkers for early stage LA diagnosis (Han et al., 2020).

*miR-1169* and *miR-260* can effectively distinguish between EGFR wild-type and mutant NSCLC patients (Xia et al., 2021). Additionally, *miR-181-5p, miR-30a-3p*, *miR-30e-3p*, and *miR-320b* have emerged as key biomarkers for differentiating LA from squamous cell carcinoma (SCC) in NSCLC, with a diagnostic accuracy demonstrated by an AUC value of 0.936 for detecting SCC (Jin et al., 2017). Furthermore, the combined application of multiple exosomal miRNAs improves the accuracy of NSCLC diagnosis. Specifically, the combination of exosomal *miR-382* and *CEA* in serum (AUC: 0.953) (Luo et al., 2021) and plasma exosomal *miR-let-7f-5p* combined with *CEA* and *CYFRA21-1* (AUC: 0.981), have notable advantages in the diagnosis of NSCLC (Wang et al., 2020).

*LINC00917* in exosomes showed stronger predictive ability for stage III/IV NSCLC (AUC: 0.907) compared to stage I/II (IUC: 0.773) (Xiong et al., 2021). LASSO regression analysis was used to screen biomarkers from exosomal lncRNAs in a large clinical population through exosomes in plasma. Then, a multi-marker diagnostic model was constructed using logistic regression, which integrates specific exosomal lncRNAs (*PGM5-AS1*, *SFTA1P*, and *CTA-384D8.35*), achieving a high prediction accuracy with an AUC of 0.97 (Wang et al., 2023). Similarly, a ncRNA profile consisting of five specific lncRNAs was found to improve the diagnosis of NSCLC with an AUC of 0.947 (Talebi et al., 2022), indicating that exosomal lncRNA patterns constructed through histological research and data analysis techniques have higher diagnostic value compared to previous single biomarkers.

One proteomic analysis revealed that plasma exosomal *MFGE8* has a high diagnostic effect in distinguishing between squamous cell carcinoma and lung adenocarcinoma, with an AUC of 0.812 (Bao et al., 2022). Another identical methodology was employed, and discovered that the concurrent utilization of AHSG, ECM1, and CEA substantially increased the diagnostic accuracy for NSCLC. Specifically, the AUC values for distinguishing NSCLC from healthy individuals were 0.938 for overall NSCLC and 0.911 for early-stage NSCLC (Niu et al., 2019).

### 5.2 TEXs as prognostic markers for NSCLC

Increasing research has shown that exosomal proteins and miRNAs are closely related with tumor progression, highlighting that exosome can be utilized as prognostic markers to enhance the treatment options available for NSCLC patients (Niloufar et al., 2024). For instance, phenotypic analysis of exosomes from the plasma of 276 NSCLC patients revealed that exosomal membrane-bound proteins, such as EGFR, NY-ESO-1, ALIX, PLAP and EPCAM, are significantly associated with overall survival (OS) in patients, suggesting that exosomal membrane-bound proteins can be used as prognostic biomarkers for NSCLC (Sandfeld-Paulsen et al., 2016).

In addition, the downregulation of *miR-503* in NSCLC tissues, compared to non-malignant lung tissue, has been linked to advanced tumor stages and poor prognosis. Kaplan-Meier analysis further indicated worse survival outcomes in patients with lower *miR-503* expression, suggesting that *miR-503* could be a valuable prognostic biomarker for survival in NSCLC patients (Liu et al., 2015). Another study found that deregulated expression of *miR-21*, *miR-143*, and *miR-181a* in NSCLC is associated with clinicopathological characteristics and poor prognosis, with elevated *miR-21* expression being linked to worse survival outcomes (Gao et al., 2010).

Additionally, studies have demonstrated the potential of exosomal biomarkers such as *PLA2G10* mRNA and *RP5-977B1* lncRNA for both diagnostic and prognostic purposes in NSCLC, enhancing tumor detection, prognosis assessment, and early-stage diagnosis (Chen Yinfeng et al., 2022; Min Ling et al., 2022). Moreover, a study found that 84 plasma exosomal miRNAs from patients with LA and healthy controls and found that elevated levels of exosomal *miR-10b-5p*, *miR-21-5p and miR-23b-3p* are associated with worse overall survival, indicating that exosomal miRNAs can also be used as prognostic biomarkers for NSCLC (Liu et al., 2017). In recurrent cases of NSCLC patients, elevated levels of exosomal *miR-203-3a-3p* (Han B. et al., 2022) and *miR-124* (Sanchez-Cabrero et al., 2023) reveal the potential for exosomal miRNAs to predict disease progression.

Furthermore, studies have reported significant associations between exosomal lncRNA and the prognosis, lymph node metastasis, TNM stage, and tumor invasion (Lin Shuai et al., 2024; Yin Cunli et al., 2024; Zhang et al., 2025) in NSCLC patients. In summary, exosomes offer a promising, non-invasive approach for prognostic biomarker development in NSCLC.

### 5.3 TEXs as markers of targeted therapy resistance in NSCLC

In recent years, targeted therapy has garnered significant attention and yielded remarkable outcomes in the treatment of NSCLC patients. Nonetheless, despite an initial positive response to targeted therapy, the eventual development of acquired resistance is inevitable, resulting in deteriorated treatment outcomes and prognosis. Consequently, it is imperative to unravel the fundamental mechanisms underlying targeted resistance and identify potential biomarkers and targets that contribute to the resistance to tumor-specific targeted therapy. Increasing research suggests that exosomes can promote resistance through various mechanisms. Exosomes exhibit the ability to convey miRNA, lncRNAs, and proteins to targeted cells, facilitating the transmission of signals between resistant and sensitive cells, as well as between stromal and tumor cells, ultimately leading to the induction of drug resistance in tumor cells (Shedden et al., 2003; Bach et al., 2017; Yu et al., 2015).

Exosomal miRNAs have been shown to play a significant role in drug resistance, particularly in EGFR-TKIs. TEXs contribute to EGFR-TKI resistance by transferring active cargoes, including miRNAs. Research has demonstrated that exosomal RNA can detect EGFR-T790M and activated EGFR mutations with sensitivities of 90% and 98%, respectively (Krug et al., 2018). In addition, Nano-LC-MS/MS analysis of gefitinib-resistant PC9R cells, due to the EGFR-T790M mutation, revealed the enrichment of specific exosomal proteins (Choi et al., 2014). Extensive research has demonstrated that the level of expression of *lncRNA H19* is elevated in gefitinib-resistant NSCLC. Specifically, *lncRNA H19* is encapsulated within exosomes, facilitated by the mediation of hnRNPA2B1, and transmitted to non-resistant NSCLC cells to induce gefitinib resistance (Lei et al., 2018). Moreover, nine exosomal miRNAs were found to be upregulated in patients resistant to Osimertinib, providing a predicting basis for treatment response (Janpipatkul et al., 2021; Han et al., 2023; Kaźmierczak et al., 2022).

Additionally, exosomal circRNAs, such as*circ0008928* (Shi et al., 2023) and *circVMP1* (Xie et al., 2022),are upregulated in the serum of cisplatin-resistant NSCLC patients, suggesting a potential role in resistance to chemotherapy (Pérez-Ruiz et al., 2020). Furthermore, exosomes from an ALK-TKI-resistant NSCLC subclone have been shown to induce drug resistance in a previously sensitive subclone. Differential expressions of miRNAs, including *miR-21-5p* and *miR-486-3p*, and lncRNAs like *MEG3* and *XIST* were identified in exosomes secreted by resistant subclones (Kwok et al., 2019).

These findings underscore the potential of TEXs as biomarkers for assessing the efficacy of targeted therapies through liquid biopsy. TEXs could also serve as indicators of resistance to targeted therapy in NSCLC, providing valuable insights for monitoring treatment response and predicting resistance.

### 5.4 TEXs as markers for immunotherapy in NSCLC

Immunotherapy has significantly transformed the treatment of NSCLC with immune checkpoint inhibitors (ICIs) playing a central role (Addeo et al., 2021; Li et al., 2011; Sharma and Allison, 2015). These therapies, including antibodies targeting the PD-1/PD-L1 pathway and cytotoxic T-lymphocyte-associated protein 4 (CTLA-4), have significantly improved patient outcomes, especially when combined with chemotherapy (Yu et al., 2016). These therapies, including antibodies targeting the PD-1/PD-L1 pathway and CTLA-4, have significantly improved patient outcomes, especially when combined with chemotherapy.

However, beyond PD-1/PD-L1 and CTLA-4, other immune checkpoints are emerging as important therapeutic targets. These include lymphocyte activation gene-3 (LAG-3), T cell immunoglobulin and mucin domain-containing protein 3 (TIM-3), T cell immunoreceptor with Ig and ITIM domains (TIGIT), V-type immunoglobulin domain-containing suppressor of T cell activation (VISTA), and CD276, each playing distinct roles in immune regulation and contributing to tumor immune evasion (Yin Nanhao et al., 2024). Currently, these checkpoints are under active investigation for their potential in enhancing immunotherapy responses, often in combination with existing PD-1/PD-L1 therapies. Exosome-based biomarker research in NSCLC has primarily focused on the PD-1/PD-L1 pathway, with exosomal PD-L1 demonstrating significant potential as a non-invasive marker for monitoring immunotherapy responses. While studies on exosomal PD-L1 have shown a correlation with treatment outcomes, research into other immune checkpoint markers, such as CTLA-4, is still limited.

**FIGURE 3:**
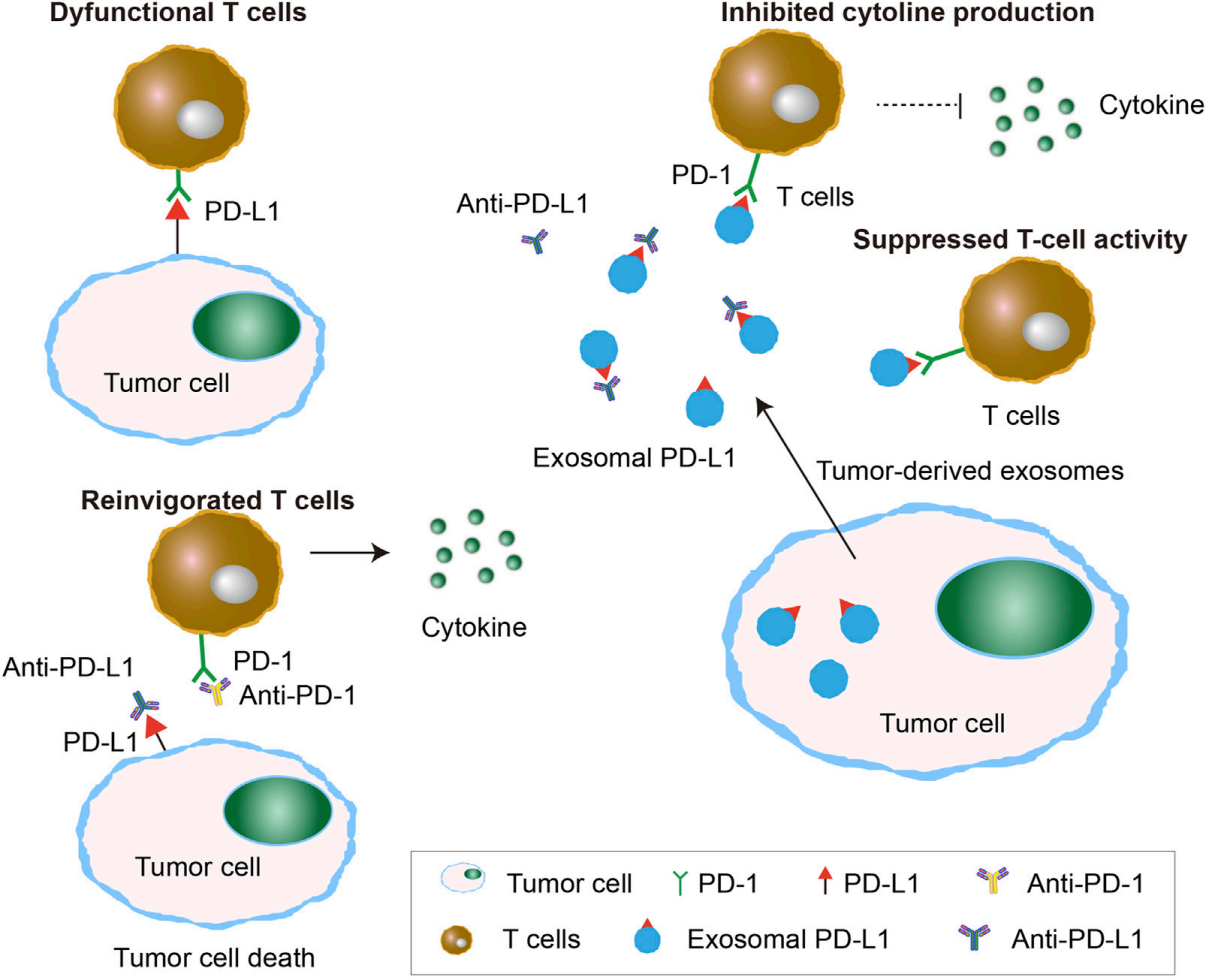
Exosomal PD-L1 caused failure of immune check-point therapy. Exosomal PD-L1 released by tumor cells causes the failure of immune checkpoint therapy by binding to PD-1 receptors on T cells, leading to suppressed T-cell activity and inhibited cytokine production. This prevents T-cell reinvigoration despite the use of anti-PD-1/PD-L1 therapies, allowing the tumor to evade immune detection.

PD-L1 is a key protein in tumor cells that binds to the PD-1 receptor on T cells, inhibiting their activation and promoting immune evasion by suppressing T cell activity. This interaction allows tumor cells to escape immune surveillance, making it harder for the immune system to attack them (Xia et al., 2019). PD-L1 is present not only on the surface of numerous tumor cell types, but also on the surface of exosomes, known as exosomal programmed death-ligand 1 (exo-PD-L1) (Ayala-Mar et al., 2021). Tumor-derived Exo-PD-L1 has the capability to competitively interact with PD-1 receptors present on the surface of T cells, inhibiting T cell activity and cytokine release, thereby mediating immune escape of tumor cells and the efficacy of immunotherapy (Figure 3). A study involving 85 patients with NSCLC demonstrated a significant correlation between the expression of exo-PD-L1 in serum and key clinical parameters, including tumor size, lymph node status, metastasis, and disease progression, highlighting its potential as a clinically relevant biomarker for NSCLC management (Li et al., 2019). Additionally, Peng et al. suggested that high levels of exosomal *miR-320d*, *miR-320c*, and *miR-320b* were associated with poor response to anti-PD-1 treatment in NSCLC patients, while exosomal *miR-125b-5p* was identified as a potential target for improving the effectiveness of anti-PD-1 therapy (Peng et al., 2020a).

CTLA-4, a negative regulatory receptor on effector and regulatory T cells, suppresses T cell activity and allows tumor cells to evade immune detection. The CheckMate-227 trial showed that simultaneous blockade of PD-1 and CTLA-4 significantly improved overall survival in NSCLC patients (Théry et al., 2018). In addition, a study aimed at elucidating the prognostic relevance of exo-PD-L1 and CD28 in NSCLC patients subjected to ICI treatment uncovered that patients with elevated exo-PD-L1 expression coupled with reduced CD28 levels displayed a shorter progression-free survival, underscoring the importance of considering baseline exo-PD-L1 and CD28 levels as potential prognostic indicators for the outcomes of PD-1-based therapeutic interventions (Zhang C. et al., 2020).

## 6 Conclusion

Exosomes function as pivotal “messengers” among cells, efficiently facilitating the transfer of critical signals and substances, thus enhancing intercellular communication. TEXs play a crucial role in almost every step of the invasion and metastasis process in NSCLC, such as immune regulation, angiogenesis, drug resistance, EMT, and pre-metastatic niche formation. By coordinating these complex interactions, exosomes significantly influence the progression and distant metastasis of NSCLC, highlighting their importance in the biology and dynamics of the tumor microenvironment. Moreover, the structural integrity of their lipid bilayer ensures stability both *in vivo* and *in vitro*, while protecting enclosed bioactive molecules, further supporting their potential as clinical diagnostic and prognostic tools (Kimiz-Gebologlu and Oncel, 2022).

Despite these promising attributes, the clinical translation of exosome-based liquid biopsies and therapeutics faces several key challenges. The lack of standardized and scalable isolation methods results in inconsistent purity and recovery, necessitating the development of cost-effective, high-throughput technologies with robust quality control measures. Additionally, the heterogeneity of exosomes and the complexity of their cargo complicate the identification of tumor-specific biomarkers, emphasizing the need for advanced single-vesicle analysis and omics-driven approaches. Furthermore, the limited sensitivity and specificity of exosome-based assays for early cancer detection require large-scale validation studies to establish reliable biomarker panels. The current infrastructure of conventional clinical laboratories is insufficient to handle the analytical demands of exosomal data, highlighting the need for automated and user-friendly platforms. Lastly, regulatory and logistical barriers, such as the lack of clear guidelines and extensive approval processes, delay the widespread adoption of exosome-based applications. Currently, the utilization of exosomes in the diagnostic and therapeutic of NSCLC remains in its nascent stage. Anticipated advancements in exosome research encompassing their biosynthesis, secretion processes, interactions with targeted cells, and the functional significance of exosomal constituents, have the potential to enhance their application in medical practice and elevate the survival prospects for patients afflicted with NSCLC.
